# First evidence of *Halomicronema metazoicum* (Cyanobacteria) free-living on *Posidonia oceanica* leaves

**DOI:** 10.1371/journal.pone.0204954

**Published:** 2018-10-01

**Authors:** Nadia Ruocco, Mirko Mutalipassi, Antonino Pollio, Susan Costantini, Maria Costantini, Valerio Zupo

**Affiliations:** 1 Department of Biology and Evolution of Marine Organisms, Stazione Zoologica Anton Dohrn, Villa Comunale, Napoli, Italy; 2 Department of Biology, University of Naples Federico II, Complesso Universitario di Monte Sant’Angelo, Via Cinthia, Napoli, Italy; 3 Bio-Organic Chemistry Unit, Institute of Biomolecular Chemistry-CNR, Pozzuoli, Naples, Italy; 4 Center of Villa Dohrn Ischia-Benthic Ecology, Department of Integrative Marine Ecology, Stazione Zoologica Anton Dohrn, P.ta S. Pietro, Ischia, Naples, Italy; 5 Istituto Nazionale Tumori—IRCCS—Fondazione G. Pascale, Napoli, Italy; Università della Calabria, ITALY

## Abstract

Cyanobacteria contribute to the ecology of various marine environments, also for their symbioses, since some of them are common hosts of sponges and ascidians. They are also emerging as an important source of novel bioactive secondary metabolites in pharmacological (as anticancer drugs) and biotechnological applications. In the present work we isolated a cyanobacteria in a free-living state from leaves of the seagrass *Posidonia oceanica* leaves. This newly collected strain was then cultivated under two laboratory conditions, and then characterized by combining morphological observation and molecular studies based on 16S rRNA gene sequences analysis. The strain showed 99% pairwise sequence identity with *Halomicronema metazoicum* ITAC101, never isolated before as a free-living organisms, but firstly described as an endosymbiont of the Mediterranean marine spongae *Petrosia ficiformis*, under the form of a filamentous strain. Further studies will investigate the actual role of this cyanobacterium in the leaf stratum of *P*. *oceanica* leaves, given its demonstrated ability to influence the vitality and the life cycle of other organisms. In fact, its newly demonstrated free-living stage, described in this study, indicate that *Phormidium*-like cyanobacteria could play important roles in the ecology of benthic and planktonic communities.

## Introduction

Cyanobacteria (*Cyanoprokaryota*/*Cyanophyta*) are the most ancient and dominant groups of photoautotrophic/photosynthetic and Gram-negative organisms, which played a major role in the evolution of the plant kingdom and Earth’s atmosphere. These organisms (blue-green algae) are ubiquitous in nearly all ecosystems and found as unicellular or colonial species. They resemble both bacteria and algae, exhibiting features linking them to both groups [1]. Cyanobacteria are characterized by widespread distribution in both aquatic and terrestrial ecosystems, and they are disseminated in areas ranging from hot springs to the Arctic and Antarctic regions [2–8], also playing important roles in other extreme environmental conditions, such as high salinities, pH and irradiances [9–12]. Cyanobacteria are most important biomass producers and play globally a significant role in biogeochemical cycle of nitrogen, carbon and oxygen [13]. They represent an ecologically important source of natural biofertilizers for rice-growing countries and other N_2_-deficient habitats because of their capabilities to fix atmospheric nitrogen in aerobic conditions through the nitrogenase enzyme [14–15]. Cyanobacteria depend on solar energy for important energy-dependent processes such as photosynthesis and nitrogen fixation [16]. Several species of cyanobacteria are known to produce hydrogen as a renewable and commercially feasible alternative source of energy [17]. Production of molecular hydrogen by cyanobacteria may be a striking substitute over other conventional hydrogen production processes, as it is an eco-friendly source of energy. Cyanobacteria can be transformed by gene alteration to produce ethanol with high efficiency [18]. Traditionally cyanobacteria are also used as a food supplement [19].

Since they perform photosynthesis, they contribute to the primary production in aquatic environments, releasing free oxygen. In addition, since they are able to fix nitrogen in aerobic conditions, they become suitable to establish symbiotic relationships with lichens, plants, various protists, providing energy for the host. In the marine environment the association between cyanobacteria and sponges is well-known, mainly found in *Demospongiae* and *Calcarea* families [20–21].

Moreover, these prokaryotes are excellent sources of a wide range of biologically active compounds with pharmaceutical and biotechnological applications, such as anticancer drugs, foods, feeds, fuels, and pigments as fluorescent probes and in application aimed at combating pollution [22–27].

In the present study we isolated for the first time a cyanobacterium from the leaf stratum of the seagrass *Posidonia oceanica*, in a free-living state. This cyanobacterium was massively cultivated under laboratory conditions, and then characterized by combining morphological observations under light microscopy and molecular studies based on 16S rRNA gene sequences analysis.

## Materials and methods

### Ethics statement

This study did not involve endangered or protected species.

### Strain origin and growth conditions

Leaves of *Posidonia oceanica* were collected in the meadow of Lacco Ameno (Ischia, Gulf of Naples, where this seagrass is widely distributed; Bay of Napoli, Italy: 40°44′56″ N, 13°53′13″ E) in spring (April) by scuba divers at 5 meters depth. The sampling area is included in a marine protected area and any source of anthropogenic disturbances may be excluded besides normal touristic activities. Twenty two adult leaves were collected by divers, cutting ten centimeters over the ligule with metal forceps, kept in sea water and immediately transferred to the laboratory in aerated closed-cycle tanks containing sterilized water up to the examination. In the collection site the water temperature ranged between 18.5 and 20.5°C at the time of the collection. All the leaves were individually moved to glass dishes to be examined in both sides under stereomicroscope to detect the presence of cyanobacterium filaments. The small portions of thalli that appeared homogeneous in these epiphytes were collected by forceps and moved into multi-well plates containing Guillard’s *f/2* medium (Sigma Aldrich, Milan, Italy) to follow their development during axenic growth in thermostatic chambers, at 18°C and 12/12 dark/light cycle. When pure thalli were obtained, they were further transferred every 2–3 days in multi-wells containing fresh media, up to a complete isolation. The identification of the isolated strains was performed under a light microscopy.

### Growth tests

The growth rates of pure strains were tested in two culture media: Guillard’s f/2 (Merck, Milan, Italy) and BG-11 medium for cyanobacteria (Merck, Milan, Italy). In both cases, the strains were kept in a thermostatic chamber at 18°C with a 12/12 dark/light photoperiod. To this end, small portions of cyanobacteria thalli were collected, with an initial average weight comprised between 170 and 400 mg (Drained Fresh Weight). Twelve replicate samples were distributed into multi-well plates containing 10 ml of culture medium each, in axenic conditions, for each of two treatments (consisting in the two culture media above defined). Every 5 days the fresh weight was recorded for each replicate in each of the two treatments. The culture medium was replaced in all the wells after 15 days. After 25 days, the average percent daily production was evaluated in order to compare the effect of the two media on the growth and persistence of this species.

### Morphological identification

Small pieces of matte were collected and inoculated in Guillard’s *f/2* (Sigma Aldrich, Milan, Italy) medium and incubated at 25°C, under continuous light supplied by Osram fluorescent tubes 36 W that provided an irradiation of 60 μE m^2^ sec^-1^. The isolation of the non-axenic unialgal strains from the matte was obtained by furhter transfers of single filaments, performed using sterilized lancets under a stereo microscope (Leica M165 C). Isolates were maintained both in a liquid and a solid medium (agar 1,5%). The strain was examined using light microscopy (LM) with a Nikon Eclipse 800 Optical microscope equipped with objectives Plan–Apochromat 100×/1.4 N.A., oil immersion, DIC). Images were captured using a DXM1200F digital camera and ACT-1 software (Nikon USA). The morphological characters observed were: filament and cell shape and dimensions, shape of terminal cell, presence of calyptra, sheaths, type of reproduction.

### DNA extraction

Freshly growing culture (about 400 mg) was homogenized and disrupted under liquid nitrogen. DNA was extracted according a method previously described by Singh et al. [28]. Briefly, 400 μl of lysis buffer (Urea 4 M; Tris–HCl 0.2 M, pH 7.4; NaCl 20 mM and EDTA 0.2 M) and 50 μl of Proteinase K (20 mg/ml) were added to the sample and then incubated for 1 hour at 55°C. Then, 1 ml of prewarmed DNA extraction buffer (CTAB 3%; NaCl 1.4 M; EDTA 20 mM; Tris–HCl 0.1 M, pH 8.0; Sarkosyl 1% and Mercaptoethanol 1%)was added and newly incubated for 1 hour at 55°C. DNA was extracted by adding 2 volumes of chloroform: isoamyl alcohol (24:1 v/v), precipitated with 2 volumes of 100% ethanol and 0.1 volume of 3 M sodium acetate (pH 5.2) for 1 hour at -20° C. After centrifugation at 10000 x g for 3 minutes, precipitated DNA was washed with 70% ice-cold ethanol and dried pellets were stored in 50 μl of sterile water.

The genomic DNA concentration was quantified by NanoDrop spectrophotometer (ND-1000 UV–vis Spectrophotometer; NanoDrop Technologies, Wilmington, DE, USA). The integrity of DNA was evaluated by electrophoresis on 0.8% agarose gel.

### 16S rRNA gene sequence analysis

The gene coding for 16S rRNA subunit was amplified by polymerase chain reaction (PCR) using two cyanobacteria-specific universal primer pairs:

- forward primer CYA106F with an equimolar mixture of reverse primers CYA781R(a) and CYA781R(b) [29], amplifying a fragment of 634 bp.
- primers 27F and 23S30R [30] to amplify the 16S-23S intergenic segment of 1800 bp.

PCR reactions were performed in a 30 μl reaction mixture containing approximately 5 ng of genomic DNA, 10x PCR Reaction Buffer (Roche, Milan, Italy), 10x dNTPs (2 mM), 50 pmol/μl of each primer, 5 U/μl Taq Polymerase (Roche, Milan, Italy) as follows: initial denaturation at 95°C for 5 min; [35 cycles of denaturation at 95°C for 1 min, annealing at 60°C for 1 min and extension at 72°C for 1 min for CYA106/CYA781^(a,b)^] or [35 cycles at 95°C for 30 sec, 55°C for 30 sec and 72°C for 2 min for 27F/23S30R]; final extension at 72°C for 10 min.PCR products were separated by 1.5% agarose gelelectrophoresis in TAE buffer (40 mM Tris–acetate,1 mM EDTA, pH 8.0) and purified using QIAquick Gel Extraction Kit (Qiagen) according to the manufacturer’s instructions.

PCR products were subjected to DNA sequencing of both strands. The total 16S region was aligned to Gene Bank using Basic Local Alignment Search Tool (BLAST; [31]) and then aligned with highly similar sequence using MultiAlin (http://multalin.toulouse.inra.fr/multalin/). For phylogenetic analysis thirty two 16S rRNA gene sequences of cyanobacteria have been considered (see S1 Table for specie name and their accession number). Multiple sequence alignments were conducted using the CLUSTALW program (http://www.genome.jp/tools-bin/clustalw).

Multiple sequence alignments were performed using the service named FASMA available at http://bioinformatica.isa.cnr.it/FASMA/ ([32]; S2 Table).

## Results

### Growth rate

The growth tests indicated that the average production of this strain, cultivated in the *f/2* medium is 3.306% (±1.5) of its biomass per day, while the average production in BG-11 medium is 2.022% (±1.5) of its biomass per day. The growth exhibited an initial lag at the moment of the transfer and it restarted after five days of culture. The traditional culture medium (*f/2*) elicited the best performances and the cyanobacterium demonstrated to be easily cultivated and produced in the laboratory conditions. The production appeared continuous when the medium was maintained fresh and a steady phase was never reached in our experimental conditions.

### Morphology

**Fig 1:**
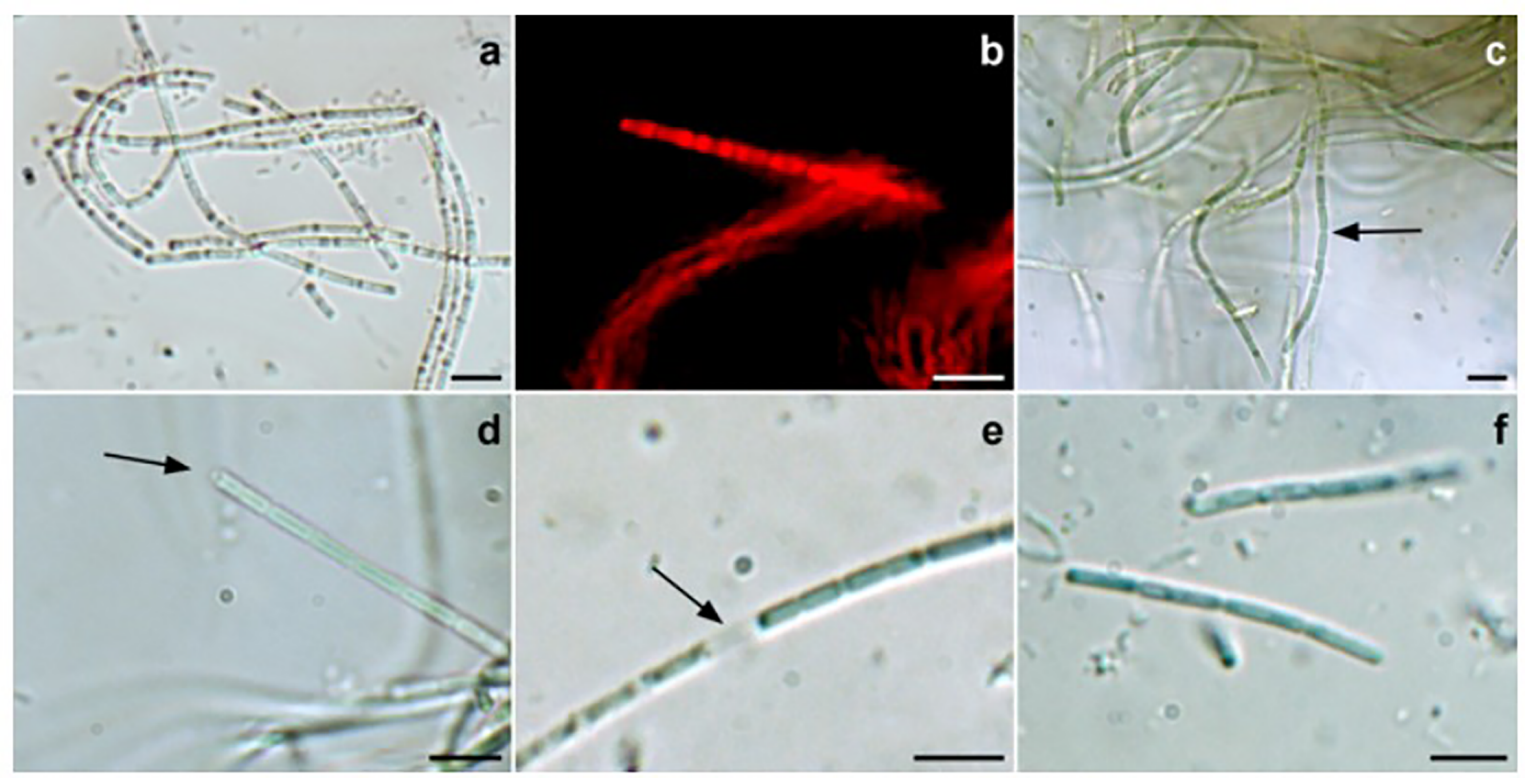
Light micrographs of *H*. *metazoicum*. A) Flexuous, fasciculated trichomes. B) Light fluorescence micrograph. Filaments are surrounded by an amorphous colorless sheath, emerging from the filaments. C) Cells of each trichome are cylindrical, with constrictions at the cross walls (arrow). D) Occurrence of a papilla (arrow) at the apex of a trichome. E) Necridic cell (arrow) between two neighbouring cells. F) Short hormogonia originated by trichome fragmentation. Bars = 5 μm.

In liquid cultures observed during the exponential phase of growth, filaments appeared as emerald-green macroscopic aggregates, which generally did not adhere to the culture vessel (Fig 1).

In old cultures the color turns from green to reddish-brown. Filaments at the LM appeared straight, or loosely and irregularly curved, with a various length, frequently entangled, rarely solitary, not motile. Sheath was colorless, generally thin, amorphous and somewhat diffused around the trichome, but rarely extending past the trichome apex. A calyptra was evident at the end of several filaments. Cells were 0.8–1.0 μm in diameter, cylindrical, elongated, usually 2–5 μm long. Terminal cells were rounded, constrictions at cross wall present, gas vesicles were not visible under the light microscopy. Reproduction occurs by fragmentation in a few celled hormogonia.

### Molecular characterization

**Fig 2:**
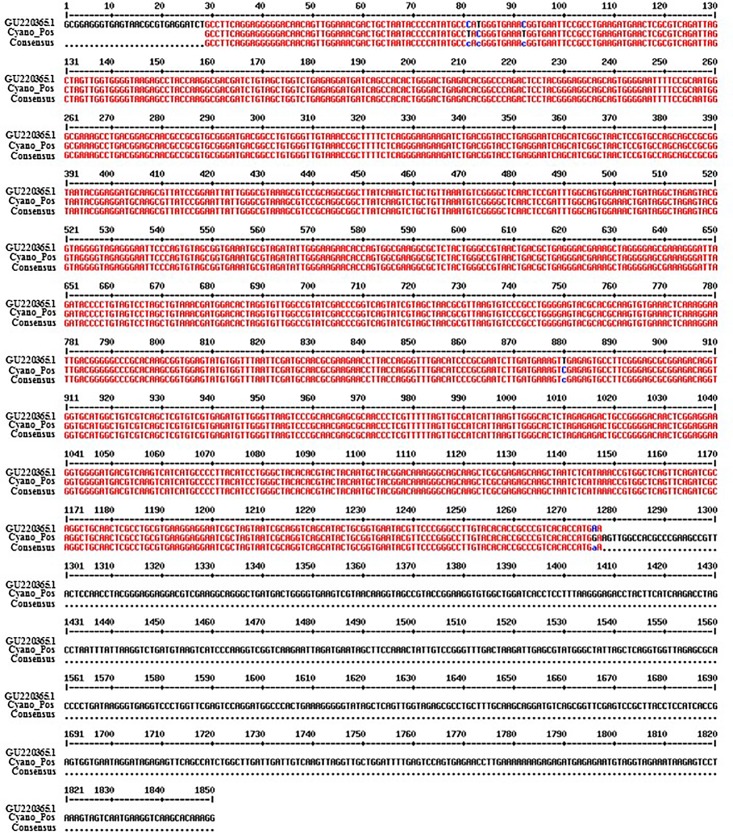
Nucleotide sequence alignment of *Halomicronema metazoicum* ITAC101 16S ribosomal RNA gene (accession number GU220365.1; retrieved from NCBI; https://www.ncbi.nlm.nih.gov/) with cyanobacterium strain isolated in this work (*Cyano_Pos*), using the software MultiAlin (http://multalin.toulouse.inra.fr/multalin/).

The cyanobacterium strain was characterized by 16 rRNA gene analysis. Our strain showed 99% pairwise sequence identity with *Halomicronema metazoicum* ITAC101 (isolated for the first time by Caroppo et al. [33]; S1 Fig and Fig 2), for which has been reported 99.9% pairwise sequence identity with *Halomicronema* sp., strain *Goniastrea*-1 isolated from the skeleton of the reef-building coral *Goniastrea aspera* (accession number GU220365.1; [34]).

**Fig 3:**
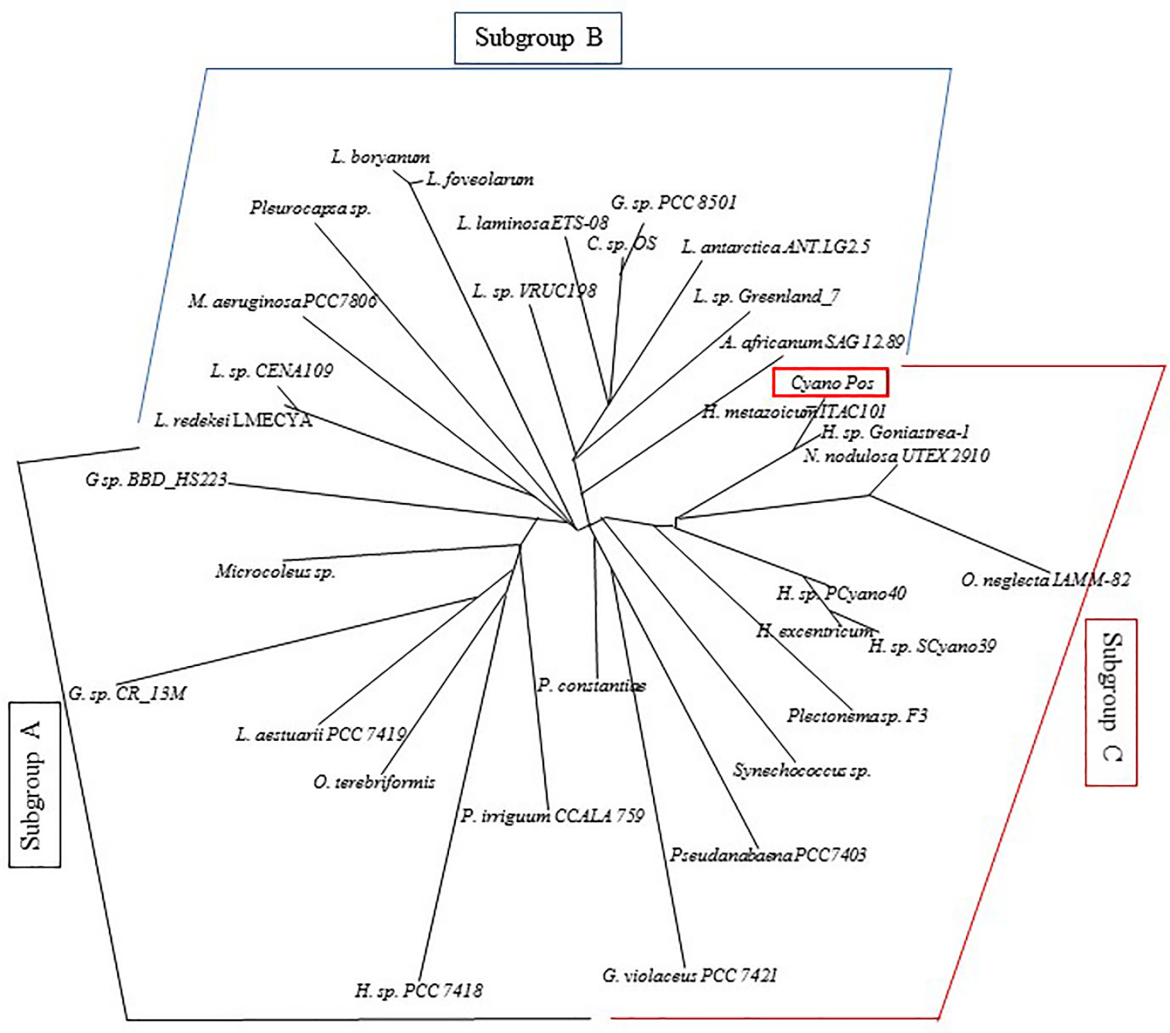
Phylogenetic tree based on 16S rRNA gene sequences. Strain isolated in this work (*Cyan_Pos*) is indicated with red box. Three subgroups are identified: subgroup A grouped with black bracket, subgroup B with light blue bracket, subgroup C with red bracket.

The two strains clustered together in a different clade of that formed by the species *Nodosolinea nodulosa* UTEX 2910 (GenBank accession number EF122600.1; Fig 3; see also S1 Table), erected as new genus able to form nodules by Perkerson et al. [35] and *Oscillatoria neglecta* IAM M-82 (GenBank accession number AB003168.1; [36]).

Phylogenetic tree showed three subgroups characterized by different species numbers and sequence mean identity percentage (Fig 3; S2 Table). More in details, subgroup A includes seven cyanobacteria species with mean sequence identity percentage of 89.7%; subgroup B includes thirteen species with mean sequence identity percentage of 89.9%; subgroup C includes thirteen species with mean sequence identity percentage of 91.6%.

## Discussion

This is the first demonstration, to our best knowledge, of *Halomicronema* cyanobacteria species living in a free state in the leaf stratum of the seagrass *Posidonia oceanica*. Based on the combination of morphological and molecular characters, the cyanobacterium *Cyano_Pos* has been attributed to the *Halomicronema* genus, family *Pseudanabaenaceae*.

Light microscopy observations revealed that this cyanobacterium showed the same morphology of that described in Carappo et al. [33]. In fact, it appeared as a filamentous strain, with thin filaments as green macroscopic aggregates. This *Halomicronema* species (reported as *Halomicronema metazoicum* ITAC101, because of the association with widespread species of marine sponges and corals) has been isolated, for the first time, in the Mediterranean Sea associated, in symbiotic relationship, to the marine sponge *Petrosia ficiformis* [33]. The morphological characterization has been supported by the molecular results based on 16S rRNA gene sequence analysis [37]. Our cyanobacterium strain *Cyano_Pos* showed 99% pairwise sequence identity with *H*. *metazoicum* ITAC101. To date, eight cyanobacterial strains were isolated from this marine sponge, and among them, the strain ITAC101 was firstly ascribed to the genus *Phormidium* [38], then to the *Lyngbya*/*Plectonema*/*Phormidium*–group B [39]. Lately, they were included in the genus *Leptolyngbya* by Anagnostidis and Komárek [40]. Furthermore, ten years later the combination of molecular and traditional techniques revealed that cyanobacteria with *Phormidium*–like morphology do not represent a monophyletic group [41–42]. In fact, a new genus of non–heterocystous, thin filamentous species has been described as *Halomicronema excentricum* by Abed et al. [43]. These data were in agreement with our phylogenetic analysis (reported in the phylogenetic tree in Fig 3). In the present study we demonstrated that *Cyano_Pos* was phylogenetically distant from *Leptolyngbya* genus. Some cyanobacteria belonging to *Leptolyngbya* genus were included in the subgroup B (*Leptolyngbya* sp. 'VRUC198/Albertano 1992, *Leptolyngbya* sp. Greenland_7, *L*. *antarctica* ANT.LG2.5, *L*. *boryanum*, *L*. *foveolarum*, *L*. *laminosa* ETS-08 strain), whereas *Cyano_Pos* in the subgroup C together with some cyanobacteria belonging to *Halomicronema* genus (*Halomicronema* sp. SCyano39, *Halomicronema* sp. PCyano40, *Halomicronema* sp. TFEP1, *Halomicronema* sp. Goniastrea-1 gene, *H*. *metazoicum* ITAC101). Moreover, *H*. *metazoicum* ITAC101, and consequently our *Cyano_Pos* strain, resulted phylogenetically well-separated from *H*. *excentricum* (with a 93.5% pairwise sequence identity; see S2 Table), forming a distinct sister clade in the subgroup C.

Our findings supported the need to combine morphological and molecular analyses to describe microbial Cyanobacteria species, mainly for the identification of cyanobacterial strains both at genus and at species level [44]. In fact, molecular and biochemical techniques are greatly expanding scientific knowledges on the ecological role and the biodiversity of cyanobacteria [45].

An important insight of this study was the discovery of a free-living *H*. *metazoicum*, considering that these cyanobacteria were known to live in symbiotic association with various metazoans. In addition, to date, several cyanobacteria may live in association with taxonomically diverse organisms including animals, plants, fungi, algae, non-photosynthetic protists, and bacteria, both in terrestrial and marine environment.

The first examples were reported from terrestrial environments. West and Adams [46] isolated forty free-living cyanobacteria from the hornwort *Phaeoceros laevis*: two were *Calothrix* spp., three were *Chlorogloeopsis* spp. and the rest were *Nostoc* spp. Free-living *Nostoc* cyanobacteria were very frequently found on terrestrial plants as reviewed in Meeks and Elhai [47]. Later, a free-living chlorophyll d-producing cyanobacterium, *Acaryochloris marina* MBIC-11017, has been discovered in the eutrophic hypersaline lake Salton Sea [48]. N_2_-fixing free-living heterocystous cyanobacteria (*Nostoc*, *Calothrix*) have has been also found in some southern California streams spanning broad nutrient gradients, indicating that their abundance is a good indicator for a rapid nutrient biomonitoring [49]. Free-living *Calothrix rhizosoleniae* and *Richelia intracellularis* have been found in the South China Sea and their abundance increased with a deepening nitracline and increasing surface seawater temperature [50].

*Halomicronema* species was never reported as a free-living cyanobacterium in the marine environment. This species has haemolytic activity and it is able to influence brine shrimp vitality and sea urchin embryo development [51–52]. Aqueous extracts from eight cyanobacterial strains isolated from the Mediterranean sponge *Petrosia ficiformis* have been investigated for their bioactive properties [52]. Some aqueous extracts, obtained from isolated cyanobacterial strains, belonging to *Leptolyngbya* and *Synechococcus* genera (to which *Cyano_Pos* belongs), exhibited citolytic effect on human erythrocytes and toxic activity against *Artemia salina nauplii*. Furthermore, antimitotic activity has been found during the development of sea urchin *Paracentrotus lividus* embryos and disorganization of blastomeres with altered cell-cell contact. These data permit to forecast that the cyanobacteria to which *Cyano_Pos* belongs represents a possible source of novel bioactive [53], even with potential applications in the pharmaceutical field [54].

Further studies will investigate the actual role of this cyanobacterium when they are free-living in *P*. *oceanica* leaves. In fact, their possible presence in both planktonic and benthic communities, even in extreme conditions [55] demonstrated by the free living form just discovered, indicates that exudates produced by this genus of microorganisms [56] may have direct effects on the life of various associated organisms. Therefore, the identification of this free-living form claims for the need of further investigations aimed at defining its actual ecological roles in seagrass environments, similarly to what already demonstrated for other cyanobacteria in marine environments [57]. In fact, this study is very interesting especially because the understanding of the ecological role of these organisms in the nitrogen fixation in *P*. *oceanica* meadows can help to better understand the nutrient cycling in this ecosystem so complex and so relevant for the biogeochemistry of coastal waters. Finally, it is also important to consider the potential of marine cyanobacteria as anticancer agents and their possible biotechnological applications [58–59] for their secondary metabolites.

## Supporting information

S1 Table Specie names, acronyms (used in the phylogenetic tree of Fig 3) and accession numbers of cyanobacteria used for phylogenetic analysis of 16S rRNA gene sequences. (DOCX)

S2 Table Sequence identity percentage of the thirthy four cyanobacteria species used for phylogenetic tree. (XLS)

S1 Fig Top Blast Hit of 16S rRNA gene sequence of cyanobacterium isolated in this work on the *Posidonia oceanica* leaves by Basic Local Alignment Search Tool (BLASTN; https://blast.ncbi.nlm.nih.gov/Blast.cgi). (JPG)
