# Supplementary material for: First evidence of Halomicronema metazoicum (Cyanobacteria) free-living on Posidonia oceanica leaves
Source: PLoS One. 2018 Oct 1;13(10):e0204954. doi: 10.1371/journal.pone.0204954 (PMC6166977; doi:10.1371/journal.pone.0204954)
Supplement: S1 Table — (DOCX) [file pone.0204954.s001.docx]

**S1 Table.** Specie names, acronyms (used in the phylogenetic tree of Fig. 3) and accession numbers of cyanobacteria used for phylogenetic analysis of 16S rRNA gene sequences.

| **Name** | **Acronym** | **Accession number** |
| --- | --- | --- |
| *Arthronema africanum SAG 12.89* | *A. africanum SAG 12.89* | AB115966.1 |
| *Cyanobacterium sp. (OS type I)* | *C. sp. OS* | L04709.1 |
| *Geitlerinema sp. BBD_HS223* | *G sp. BBD_HS223* | DQ680351.1 |
| *Geitlerinema sp. CR_13M* | *G. sp. CR_13M* | EF545604.1 |
| *Geitlerinema sp. PCC 8501 strain PCC 8501* | *G. sp. PCC 8501* | FM210758.1 |
| *Gloeobacter violaceus PCC 7421* | *G. violaceus PCC 7421* | AF132790.1 |
| *Halomicronema sp. SCyano39* | *H. sp. SCyano39* | DQ058860.1 |
| *Halomicronema sp. PCyano40* | *H. sp. PCyano40* | DQ058890.1 |
| *Halomicronema excentricum TFEP1* | *H. excentricum* | AF320093.1 |
| *Halomicronema sp. Goniastrea-1 gene* | *H. sp. Goniastrea-1* | AB257773.1 |
| *Halomicronema metazoicum ITAC101* | *H. metazoicum ITAC101* | GU220365.1 |
| *Halothece sp. PCC 7418* | *H. sp. PCC 7418* | AF296872.1 |
| *Leptolyngbya sp. 'VRUC198/Albertano 1992'* | *L. sp. VRUC198* | DQ295207.1 |
| *Leptolyngbya sp. Greenland_7* | *L. sp. Greenland_7* | DQ431002.1 |
| *Leptolyngbya antarctica ANT.LG2.5* | *L. antarctica ANT.LG2.5* | AY493603.1 |
| *Leptolyngbya boryanum* | *L. boryanum* | X84810.1 |
| *Leptolyngbya foveolarum* | *L. foveolarum* | X84808.1 |
| *Leptolyngbya laminosa strain ETS-08* | *L. laminosa ETS-08* | FM210757.1 |
| *Limnothrix sp. CENA109* | *L sp. CENA109* | EF088335.1 |
| *Limnothrix redekei LMECYA 145* | *L. redekei LMECYA 145* | EU078512.1 |
| *Lyngbya aestuarii PCC 7419* | *L. aestuarii PCC 7419* | AB075989.1 |
| *Microcystis aeruginosa PCC7806* | *M. aeruginosa PCC7806* | U03402.1 |
| *Microcoleus sp.* | *Microcoleus sp.* | X70770.1 |
| *Nodosolinea nodulosa UTEX 2910* | *N. nodulosa UTEX 2910* | EF122600.1 |
| *Oscillatoria neglecta IAM M-82* | *O. neglecta IAM M-82* | AB003168.1 |
| *Oscillatoria terebriformis* | *O. terebriformis* | AF263343.1 |
| *Phormidium cf. irriguum CCALA 759* | *P. irriguum CCALA 759* | EU196638.1 |
| *Plectonema sp. F3* | *Plectonema sp. F3* | AF091110.1 |
| *Pleurocapsa sp.* | *Pleurocapsa sp.* | X78681.1 |
| *Pseudanabaena PCC7403* | *Pseudanabaena PCC7403* | AB039019.1 |
| *Pseudanabaena constantiae* | *P. constantiae* | DQ393595.1 |
| *Synechococcus sp.* | *Synechococcus sp.* | D88288.1 |
