# Supplementary figures and images for: First evidence of Halomicronema metazoicum (Cyanobacteria) free-living on Posidonia oceanica leaves
Source: PLoS One. 2018 Oct 1;13(10):e0204954. doi: 10.1371/journal.pone.0204954 (PMC6166977; doi:10.1371/journal.pone.0204954)

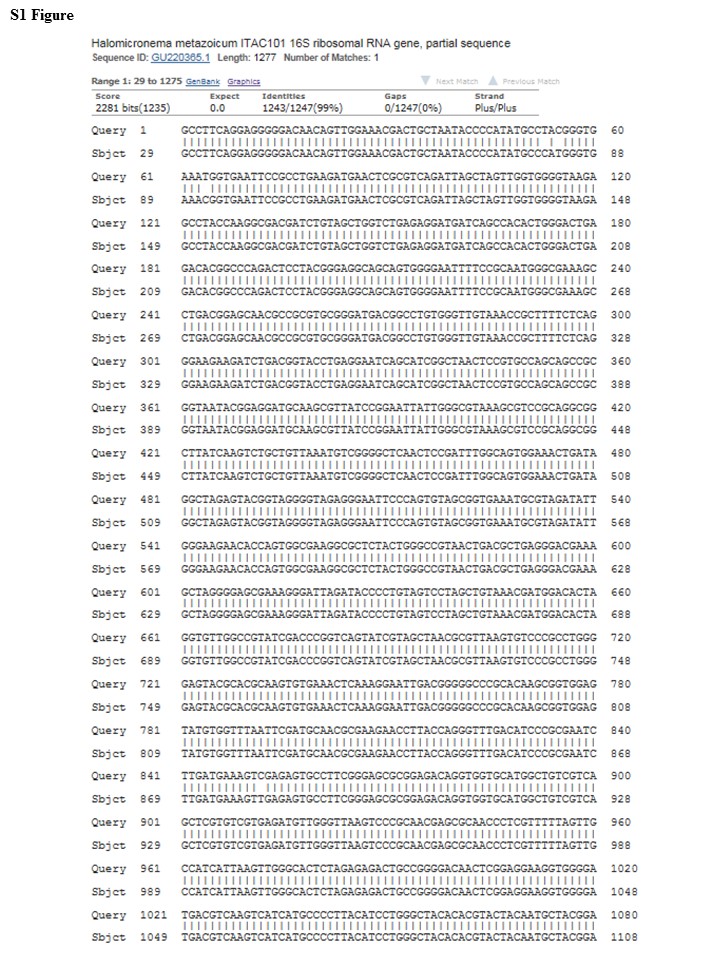

Supplement: S1 Fig — (JPG) [file pone.0204954.s003.jpg]
